# Dr. George H. Wilson

**Published:** 1922-07

**Authors:** 


### OBITUARY

### Dr. George H. Wilson

Dr. George H. Wilson was born at Painesville, Ohio, March 3, 1855, and died at Cleveland, Ohio, April 12, 1922.

He began practice with his father in Painesville in 1874 and proceeded from there to Ann Arbor for the years 1876-7 and 1877-8, graduating in the spring of 1878.

Following his graduation he returned to Painesville and practiced there until 1891. In the autumn of this year he moved his office to Cleveland, where he entered on his specialty of Prosthetics, in which he gained national prominence and respect.

It was also in Cleveland that he began his career as a teacher, being connected with the Homeopathic school during 1891 and leaving this school to become affiliated with the Dental Department of Western Reserve University when it was organized in 1892, and retaining this affiliation until 1906.

In 1911 his text-book, ''Manual of Dental Prosthetics,'' first appeared. The reception accorded this authoritative work is evidenced by the fact that the fourth edition was required in 1920.

In 1918 he was presented with the Jarvis Medal, and in 1921 he was elected a Fellow of the American College of Dentists. He was also the permanent Honorary President of the recently organized Society of Denture Prosthetists.

During the latter years of his life he was associated with the University of Southern California, dividing his time between Los Angeles and Cleveland.

He was married in Painesville January 1, 1880, to Kittie Cooley, and two children, Dr. Harris R. and Paul L., surive.

In the passing of Dr. Wilson the dental profession has lost a scientist, a scholar, an author and a teacher; many members of the profession have lost a friend, and society at large has lost a gentleman. To those who knew him best these attributes were constantly present. His devotion to his profession was most noteworthy and his contributions to its literature will link his name with those who have done most for its advancement.

To attempt to eulogize him to his friends is futile. Kind, gentle, firm, courteous, generous, fearless—so he lived and so he died.

To have but known him is a rich inheritance.

---

The following resolutions were adopted by the Dental College Faculty of Western Reserve University as an appreciative memorial of Dr. Wilson's life work:

RESOLUTIONS ADOPTED BY THE FACULTY OF THE DENTAL COLLEGE, WESTERN RESERVE UNIVERSITY

The faculty of the Dental College of the Western Reserve University, having learned of the sudden passing away of Emeritus Professor George H. Wilson, on April 12th, desire to express its great loss and sympathy. Be it therefore

*Resolved,* That the death of Professor Wilson having occurred at the zenith of his great professional career, and with much work in preparation, his untimely demise has been a distinct loss to the Dental Profession.

*Furthermore,* The Western Reserve University owes much to his efforts and assistance toward the founding of the Dental College in 1892, and his continuous service as Professor of Prosthesis and Metallurgy over 12 years. Subsequently, as Emeritus Professor, he lectured and gave freely of his knowledge and advice.

As a friend of the College and its students, the University suffers an irreparable loss.

*Finally,* It is the will and order of the faculty that these resolutions, with our sincerest sympathy, be sent to the family of the deceased, spread upon the minutes, and published in the dental journals

W. H. Whitslar,
F. M. Casto,
F. C. Waite,
*Committee.*

